# Statistics of Medical Schools

**Published:** 1847-04

**Authors:** 


ARTICLE VII.

### STATISTICS OF MEDICAL SCHOOLS.

The following statistics of the number of students and graduates of medical colleges for the last session, have been gleaned from various sources of information.

| | No. Stud'ts. | Graduates. |
|---|---|---|
| Jefferson Medical College, Phil'a. - - | 493 | 181 |
| University of Pennsylvania, - - - | 411 | |
| Pennsylvania College, - - . - | 95 | 34 |
| Franklin Medical College, - - - - | | 5 |
| University of the City of N. Y. - - | 410 | 123 |
| College of Physicians and Surgeons, N. Y. | | 51 |
| University of Louisville, - - - - | 349 | 75 |
| Transylvania University, - - - | 205 | 62 |
| Medical College of Ohio, - - - | 170 | |
| Cleveland College, Ohio, - - - | 216 | |
| Willoughby Medical College, Ohio, - - | 101 | 38 |
| Rush Medical College, Chicago, Ill., - | 70 | 17 |
| Castleton Med. College, Vt. - - - | 131 | 42 |
| Med. Dep't. of Yale College, Conn. - - | | 28 |
| Memphis Med. College, - - - - - | 55 | |
| Indiana Med. College, - - - - - | | 19 |

